# Application of terpyridyl ligands to tune the optical and electrochemical properties of a conducting polymer

**DOI:** 10.1039/c8ra05333b

**Published:** 2018-08-20

**Authors:** Grzegorz Lisak, Klaudia Wagner, Jonathan E. Barnsley, Andrei Veksha, Gregory Huff, Anastasia B. S. Elliott, Paweł Wagner, Keith C. Gordon, Johan Bobacka, Gordon G. Wallace, Ari Ivaska, David L. Officer

**Affiliations:** Johan Gadolin Process Chemistry Centre, Laboratory of Analytical Chemistry, Åbo Akademi University FIN-20500 Åbo-Turku Finland; School of Civil and Environmental Engineering, Nanyang Technological University 50 Nanyang Avenue Singapore 639798 Singapore; Nanyang Environment and Water Research Institute 1 Cleantech Loop, CleanTech Singapore 637141 Singapore; ARC Centre of Excellence for Electromaterials Science, Intelligent Polymer Research Institute, University of Wollongong NSW 2522 Wollongong Australia; Chemistry Department, University of Otago Dunedin New Zealand

## Abstract

We present a simple and effective way of using metal and metal–ligand modifications to tune the electrochemical and optical properties of conducting polymers. To that end, a polyterthiophene functionalized with terpyridine moieties was synthesized and then the resulting film's surface or bulk was modified with different metal ions, namely Fe^2+^, Zn^2+^ and Cu^2+^ and terpyridine. The modification of the terpyridine functionalized polyterthiophene film by Fe^2+^ increased the absorptivity and electrochemical capacitance of the conducting polymer, and improved its conjugation. Further modification by Zn^2+^ and Cu^2+^ resulted in dramatically different spectroelectrochemical properties of the film. Moreover, the influence of the solvents (ACN and 1 : 1 ACN : H_2_O) in conjunction with the metal ion applied for the modification was found crucial for the electrochemical and optical properties of the films.

## Abstract 2

We present a simple and effective way of using metal and metal–ligand modifications to tune the electrochemical and optical properties of conducting polymers.

## Graphical abstract

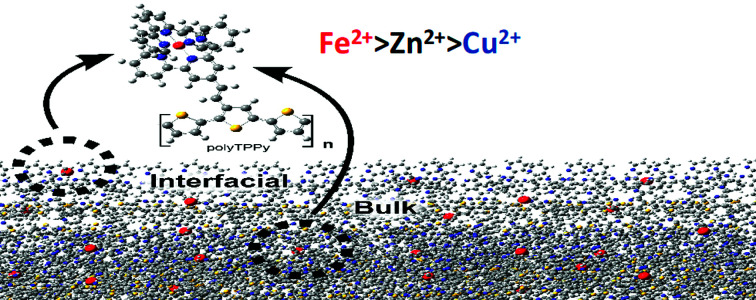

## Introduction

Conducting polymers, such as polythiophenes have been found to be suitable materials for the use in electronic, optoelectronic, photovoltaic, spin-transport studies and chemical sensor devices.^1–5^ Their usage in such a wide range of applications may be attributed to the possibility of introducing various functionalities to the thiophene, bithiophene and terthiophene monomers.^6,7^ In this respect, an interesting functional material may be obtained by introducing terpyridine substituents to the thiophene monomer. The terpyridines are effective ligands to complex metal ions, forming stable complexes consisting of two terpyridine molecules and a metal ion that are possible to be utilized in advanced molecular systems.^8–10^ The abundance of terpyridine derivatives and their ability to form stable complexes with different metal ions allows the tailoring of diverse material properties, *e.g.* obtaining cytotoxicity against several human cancer cell lines or allosterically controlling electronic effects.^11,12^ Moreover, compounds bearing the terpyridine functionality may be used to obtain different structural materials, such as molecular wires, dendrimers and hydrogels as well as polymers with terpyridine moieties in the polymer backbone or in the side chains.^13–19^ Additionally, the functionalization of conducting polymers by adding the terpyridine moiety as a side-group has led to the development of chemical sensors and tunable surfaces *via* molecular engineering and surface modifications of the metallo-organic complexes at the interface.^20–22^

The challenge in adding bulky substituents such as terpyridines to polythiophenes using functionalized thiophene monomers is the distortion in the polymer backbone that arises from neighboring substituent steric interactions. In order to overcome this, Officer and co-workers have utilized functionalized terthiophenes, although the polymerization of terthiophenes can be challenging particularly with conjugated substituents.^23–25^ The successful formation of conducting polymers from terthiophenes has however been achieved by the attachment of alkoxy substituents on the outer rings of the thiophene monomers, that not only activates the outer thiophene rings for oxidative coupling but also reduces charge localization within an aryl–aryl conjugated system that deactivates terthiophene monomer oxidative coupling.^20,26,27^

**Fig. 1:**
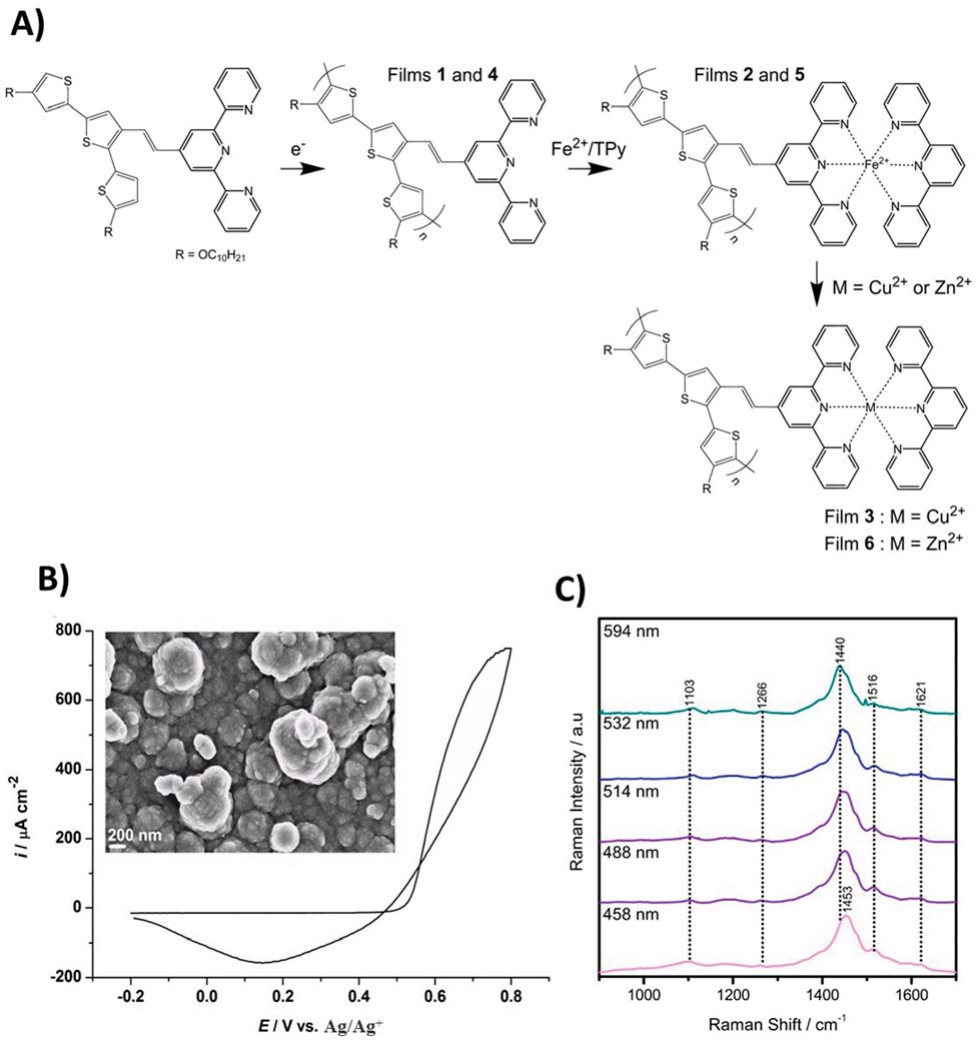
(A) Structural diagram of the preparation of poly(TTPy) films 1–6. (B) Electropolymerisation of poly(TTPy) on ITO substrate (area 1.55 cm^2^) performed by cycling the potential, during a single full cycle, between −0.2 and 0.8 V (*vs.* Ag/AgCl/0.1 M TBAP-ACN reference electrode) with a scan rate of 0.1 V s^−1^ in the solution containing 5 mM TTPy monomer and 0.1 M TBAP in 1 : 1, ACN : DCM. Inset: SEM picture of a poly(TTPy) film obtained using the same procedure for electropolymerisation but applied after 5 potential cycles. (C) Resonance Raman data for a poly(TTPy) film.

In this work, a bisdecyloxy-substituted terthiophene monomer was functionalized with a vinylterpyridine substituent to obtain (*E*)-4′-(2-(4,4′′-bisdecyloxy-2,2′:5′,2′′-terthiophen-3′-yl)ethenyl)-2,2′:6′,2′′-terpyridine (TTPy), which was subsequently modified with metal ions (Fig. 1A). Modification of the film surface and the bulk of poly(TTPy) with Fe^2+^, Zn^2+^ and Cu^2+^ metal ions afforded dramatically different optical and electrochemical film properties, with the Fe^2+^-modified film showing an increased electrochemical capacitance, better conjugation of the conducting polymer system and higher absorptivity.

## Experimental

### Electrosynthesis of poly(TTPy) and its surface modifications by metal-TPy

Cyclic voltammetry was used to electrosynthesize poly(TTPy) films on indium tin oxide (ITO) glass electrodes. The electropolymerisation was done using eDAQ system controlled by EChem software in a three electrodes system in 1 : 1 ACN : DCM solution containing 5 mM TTPy monomer with 0.1 M TBAP as supporting electrolyte. To avoid solubility problems, it was important to first dissolve the monomer in DCM while the supporting electrolyte salt (TBAP) was dissolved in ACN, followed by mixing of the two solutions to form the solution for polymerization. Before the polymerization, the ITO slide (area 1.55 cm^2^) was repeatedly washed with deionized water and ethanol (5 times) and left in ethanol for 10 min under ultrasound conditions, after which it was dried in open air. Such a pretreated ITO slide served as the working electrode. A platinum mesh electrode served as the counter electrode, while a Ag/Ag^+^/0.1 M TBAP in ACN reference electrode was used. Prior to the electrosynthesis, the reference electrode was calibrated in ferrocene solution. The electrosynthesis was done by performing one potential cycle between −0.2 and 0.8 V (*vs.* Ag/Ag^+^ l/0.1 M TBAP-ACN electrode) with the scan rate of 0.1 V s^−1^. After the polymerization the poly(TTPy) film on ITO glass was washed with ACN (films 1 and 4).

The metal complexation of the poly(TTPy) was done by first soaking the poly(TTPy) films on ITO glass for 1 h in ACN solution containing 10^−3^ M FeCl_2_. This was done in order to immobilize Fe^2+^ in mono complexes with terpyridyl moieties present in the poly(TTPy). Then the addition of 5 × 10^−3^ M TPy was done to cap the terpyridyl-Fe^2+^ mono complex with another terpyridyl group (Fe^2+^-based modifications, films 2 and 5). The films were left in the 10^−3^ M FeCl_2_ and 5 × 10^−3^ M TPy in ACN solution for 24 h. Then the films were washed with ACN and characterized by using cyclic voltammetry, optical spectroscopy, X-ray photoelectron spectroscopy (XPS) and contact angle measurement done using a goniometer. After that, the Fe^2+^-containing films were further used for subsequent modification by Cu^2+^ and Zn^2+^. The exchange of the central metal ion (from Fe^2+^ to Cu^2+^ or Zn^2+^) was done by soaking the films for 24 h either in the ACN solution containing 10^−3^ M CuCl_2_ (poly(TTPy)Cu film 3) or in the 1 : 1 ACN : H_2_O solution containing 10^−3^ M ZnCl_2_ (poly(TTPy)Zn film 6). Again, after the modification, the films were washed with ACN and characterized by using cyclic voltammetry, optical spectroscopy, XPS and contact angle measurement.

### Characterization of poly(TTPy), poly(TTPy)Fe, poly(TTPy)Cu and poly(TTPy)Zn

The thickness of the obtained poly(TTPy) film on ITO was investigated using profilometry (Dektak 150 profilometer). Field emission gun scanning electron microscopy (FEG-SEM, Leo 1530, Zeiss) was used in order to investigate the morphology of poly(TTPy). For that purpose, a thicker film was electrosynthesized as described above, but by applying five potential cycles. Resonance Raman measurements were done on the poly(TTPy) electrodeposited on ITO glass in order to confirm the conducting polymer structure. Spectra were collected using a setup which has been previously described.^28^ In short, it is composed of an excitation beam and collection lens in a 135° backscattering arrangement. Scattered photons were focused on the entrance slit of an Acton SpectraPro500i spectrograph with a 1200 grooves per mm grating, which disperses the radiation in a horizontal plane on a Princeton Instruments Spec10 liquid-nitrogen-cooled CCD detector. A Coherent Innova Sabre argon-ion laser provided 457.9, 488.0 and 514.5 nm excitation wavelengths, while solid-state CrystaLasers were used for 532.0 and 593.7 nm. Notch filters (Kaiser Optical, Inc.) or long-pass filters (Semrock, Inc.) matched to these wavelengths was used to remove the laser excitation line, and a 632.8 nm short pass filter was used to remove a sharp peak from the 593.7 nm line.

Cyclic voltammetry was performed by recording voltammograms in 0.1 M TBAP in ACN (for poly(TTPy)Cu) or in 1 : 1 ACN : H_2_O (poly(TTPy)Zn) by scanning the potential (five scans, only fifth scan shown) between 0.0 and 1.0 V (*vs.* Ag/Ag^+^/0.1 M TBAP-ACN reference electrode) with the scan rate of 0.1 V s^−1^. Optical spectroscopy was done on a dry film using a Shimadzu UV-1800 spectrophotometer (Kyoto, Japan) by recording the absorbance spectra of a film in air from 400 to 800 nm. The spectra obtained for pure ITO glass was used for background correction. For the XPS analysis the samples were cut into approximately 7 × 7 mm^2^ squares and mounted on a copper sample holder using carbon tape. The samples were grounded using silver paste connecting the ITO surface to the copper sample holder. XPS spectra were collected by illuminating the samples with a dual anode, non-monochromatic X-ray source (Omnivac) using Mg Kα radiation and the photoemission collected by a SES2002 analyzer (Scienta) operating at 200 eV (survey scan) or 50 eV (detail scan) pass energy. The spectra were processed using CasaXPS. A linear background was used, and curve fitting was carried out by restricting the width of the fitted peaks to a maximum of the resolution expected for the analyzer configuration (4 mm slit, 50 eV pass energy has a theoretical FWHM of 1 eV). The work function of the analyzer was approximately 6 eV. This and any charging shifts were accounted for by referencing the energy scale to the carbon peak at 284 eV. Quantitative results were obtained from the survey scans using the relative sensitivity factors in the CasaXPS library. No correction for analyzer transmission as a function of electron energy has been applied, which will tend to reduce the apparent concentration of the high binding energy elements by 30–40%. Finally, the hydrophobicity of all surfaces was characterized by Data-Physics OCA20 goniometer and water droplet (2 μL, Milli Q water). The contact angle was measured just after the water droplet was in contact with the measured surface. The uncertainty of the measurements was always obtained from five consecutive measurements.

### The Fe[(TTPy)_2_]^2+^ complex formation with competing ions

The Fe[(TTPy)_2_]^2+^ complex formation was investigated in presence of competing ions utilizing membrane entrapped TPPy and immobilized onto paper strips. To prepare such substrate a paper strip was dip coated in a THF solution of 1% TTPy, 0.35% KTFPB, 65.65% *o*-NPOE and 33% PVC (weight%) and left for evaporation of the solvent. Entrapment of the monomer is such matrix allowed to follow kinetically the formation of Fe[(TTPy)_2_]^2+^ complex in presence of competing ions. The measurements were performed using two different protocols. In the first protocol the measurement was done by measuring absorbance of Fe[(TTPy)_2_]^2+^ complex formation at 598 nm for 300 s at 0.1 mol dm^−3^ FeCl_2_. Then the measurements were done when Fe^2+^ was competing for complex formation with a 1 : 1 ratio of competing ions (j) to obtain the response of the Fe[(TTPy)_2_]^2+^ affected complex formation. The sequence in which the interfering ions were investigated was as follow: Na^+^, K^+^, Mg^2+^, Ag^+^, Ca^2+^, Al^3+^, Sr^2+^, Zn^2+^, Fe^3+^, NH^4+^, Ba^2+^, Mn^2+^, Hg^2+^, Ni^2+^, Co^2+^, Cu^2+^ and Ru^3+^. Each competing ion was investigated using a new Fe^2+^-SM paper strip. The influence of the competing ion on Fe^2+^ complexation by TTPy was determined then as the ratio between the maximum complex absorbances at 598 nm after 300 s of the competing ion-free and competing ion responses. In the second protocol, the absorbance spectra were taken in air from dried (approx. 1 h) paper strips used in the first protocol. The absorbance measurements were performed between 400 and 700 nm. The influence of the interfering ion on Fe^2+^ complexation by TTPy was then determined as the ratio between the Fe[(TTPy)_2_]^2+^ complex absorbance at 598 nm of competing ion-free and competing ion responses. A relatively high noise level in the absorption spectra between 450 to approx. 550 nm, attributed to the presence of paper in the measuring cell, was always observed when investigating paper-based strips.

### Computational modelling

In order to validate the obtained poly(TTPy) and its modification, geometry and vibrational calculations were generated using the Gaussian 09W program package which implemented the Becke, three-parameter, Lee–Yang–Parr (B3LYP) functional employing a LANL2DZ basis set for Fe centers and 6-31G(d) for all other atoms.^29^ No negative frequencies were observed for these optimized geometries, which indicated that local minima had been achieved. Fe^2+^ was assumed to be low spin with a singlet spin state. Cu^2+^ and Zn^2+^ were calculated with doublet and singlet spin states respectively. Formation energies (Δ*G*) were calculated for each metal complex using a simplified terthiophene unit to mimic the energetics of the larger polythiophene, while avoiding a high computational demand. While larger polymer units may have changed the overall energetics, the relative binding preferences were constrained by the local electronic and geometric nature of the terpyridine residue. Relative formation energies (Δ*G*) for each complex were calculated using a products-reactants system as shown in eqn (1)–(3):

1 Fe(MeCN)_6_^2+^ + TTPy + TPy → Fe[TTPyTPy]^2+^ + 6MeCN (for terpy binding)

2 Cu(MeCN)_4_^2+^ + TTPy + TPy → Cu[TTPyTPy]^2+^ + 4MeCN (for terpy binding)

3 Zn(MeCN)_4_^2+^ + TTPy + TPy → Zn[TTPyTPy]^2+^ + 4MeCN (for terpy binding)

Optimized energies were used for all substituents and these structures yielded no negative frequencies as above.

## Results and discussion

### Electrosynthesis and characterization of poly(TTPy)

The TTPy has been previously synthesized and used in the development of paper- and glass-based disposable iron(ii) optical sensors.^4^ In this sensor design, two properties of the monomer led to the development of novel time- and concentration-based analytical methods through, (i) the ability to complex metal ions by the terpyridyl moiety and (ii) deceleration of the complex formation by the presence of the terthiophene moiety. The Fe[(TTPy)_2_]^2+^ complex has an absorbance maximum at 591 nm, which is not masked by the absorption of TTPy complexes with any other metal ions (spectroscopic fingerprint of metal-terpyridine complex).^4^ The polymerization of TTPy, however, offered the opportunity to further expand the sensing capabilities of these species.

Conducting polymer films of poly(TTPy) (1 and 4) could be obtained by electropolymerization of the TTPy terthiophene moiety (Fig. 1A). The electrodeposition of poly(TTPy) by cyclic voltammetry is shown in Fig. 1B. A thin conducting polymeric film was obtained during a full single potential cycle. When scanning the potential from −0.2 to 0.8 V using a scan rate of 0.1 V s^−1^, the polymer film formation began at 0.55 V as a sharp increase of the current density, followed by an oxidation peak maximum around 0.8 V.

On the reverse scan from 0.8 to −0.2 V, a reduction peak was recorded at 0.15 V corresponding to the reduction of the electrodeposited poly(TTPy). Such a polymerization pattern is typical of terthiophene-based polymers,^30,31^ consequently, single CV cycles up 0.8 V were used to grow the thin polymer films (the measured thickness was approx. 95 nm). For thicker films that could be used for SEM analysis, electropolymerisation was undertaken over five consecutive potential cycles. The morphology of the film is shown in the inset of Fig. 1B. The SEM image showed full ITO coverage by the poly(TTPy) with well pronounced globular features.

**Fig. 2:**
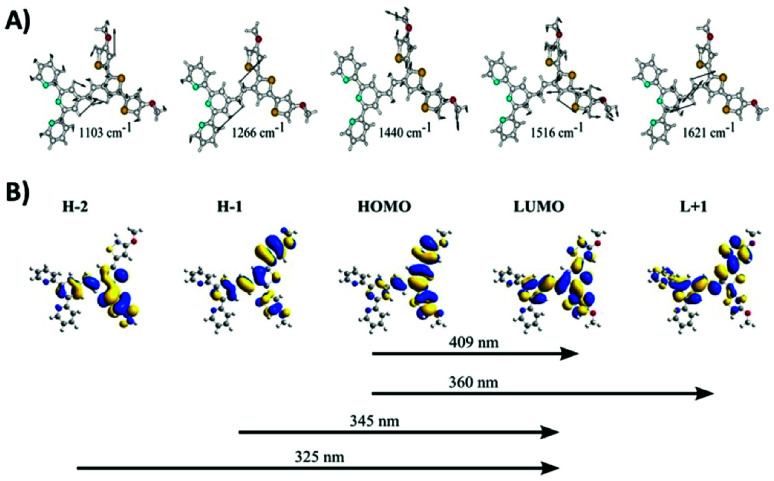
(A) DFT generated vibrational eigenvector diagrams for TPPy. (B) TD-DFT generated orbitals for a TPPy unit calculated at B3LYP/6-31G(d) level of theory.

Resonance Raman measurements were performed to characterize the poly(TTPy) film and are presented in Fig. 1C. The data showed a number of weak spectral features, which are dominated by the band at 1440–1453 cm^−1^. This band is well known as the thiophene line B and is visualized in Fig. 2A. The comparative strength of this band indicates that thiophene is involved in the transition occurring for these wavelengths. The next strongest bands at 1103 and 1516 cm^−1^, are predicted to be terthiophene based (Fig. 2A). This is consistent with electronic transitions which are terthiophene localised. Characteristic terpyridyl signals (typically around 1000, 1360, 1560 and 1590 cm^−1^) are not observed.^32^

TD-DFT data indicated this transition is of HOMO to LUMO nature, and elicits the transfer of electron density from a terthiophene orbital to an orbital delocalized over the terthiophene, C=C and terpy units (Fig. 2B). The B line vibration exhibits ‘frequency dispersion’, a redshift of band frequency as the excitation wavelength is increased, as a result of a high polarizability.^33^ This effect is uncommon in thiophene oligomers/polymers due to an increasing presence of π-stacking as the thiophene backbone is elongated; π-stacking results in a partial cancelation of π-delocalisation, despite an increased thiophene length. A ‘soft’ B line, which disperses as a function of excitation wavelength, suggests that π-stacking in this polymer film is minimal.^34^ Such a result is expected due to the steric bulk of the terpyridine residues, which may make π−π interactions between thiophene units difficult.

### Modification of poly(TTPy) with Fe^2+^

**Fig. 3:**
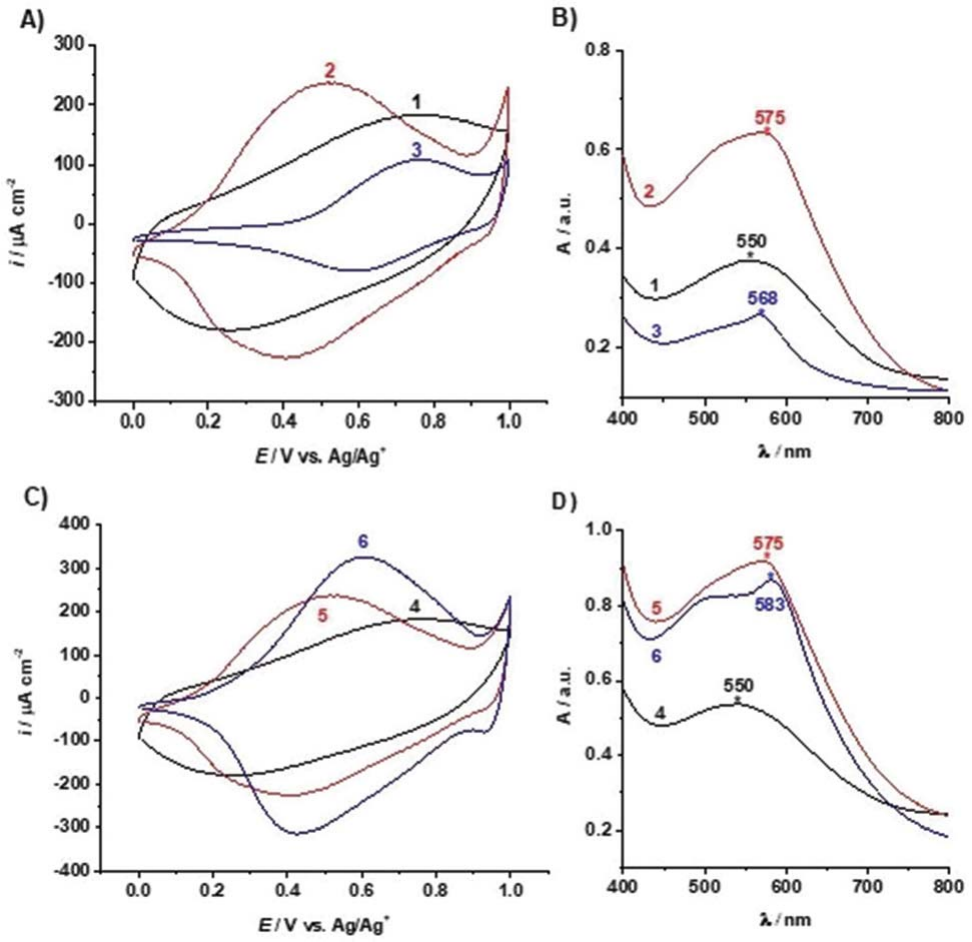
Cyclic voltammograms (fifth scan shown) performed by cycling the potential between 0.0 and 1.0 V (*vs.* Ag/Ag^+^/0.1 M TBAP-ACN reference electrode) with the scan rate of 0.1 V s^−1^ in the solution containing 0.1 M TBAP in ACN of films: (A) 1–3 and (C) 4–6. (B) UV-VIS spectra of films in air between 400 and 800 nm of films: (C) 1–3 and (D) 4–6.

The electropolymerised poly(TTPy) films on ITO glass (1 and 4) were treated with Fe^2+^ followed by TPy to give poly(TTPy)Fe films (2 and 5) (Fig. 1A). Two films were prepared in order to allow further modification with Cu^2+^ and Zn^2+^ ions. The electrochemical and optical properties of poly(TTPy) before and after Fe^2+^ modification are shown in Fig. 3A and B (films 1 and 2) and Fig. 3C and D (films 4 and 5).

From this electrochemical and optical study, it can be seen that the poly(TTPy) films 1 has a broad oxidation peak with the oxidation maximum around 0.75 V and film absorption maximum at 550 nm. The modification of poly(TTPy) film 1 by Fe^2+^/TPy (film 2) dramatically influenced its properties. The oxidation peak of the modified film was shifted towards lower potential values, namely 0.5 V, and resulted in higher electrochemical capacitance. This phenomenon indicated that the conjugation of the poly(TTPy) was improved due to the introduction of Fe^2+^/TPy to the film. This conjugation improvement may also be confirmed by the bathochromic shift in the absorbance maximum of film 2 (Fig. 3B) although, given the marked increase in absorption intensity, this must also be contributed to by the Fe[TTPy(TPy)]^2+^ complex. The metal-to-ligand charge-transfer (MLCT) band absorption for Fe[(TPy)_2_]^2+^ occurs in solution at 552 nm while that of Fe(TTPy)_2_ is substantially red shifted to 591 nm.^4^ Therefore it is not surprising that the asymmetric Fe[TTPy(TPy)]^2+^ complex would not be so red-shifted and absorb around 575 nm. Thus, it would appear that during the film modification with Fe^2+^/TPy, not only is the surface of the film modified but also the bulk of the conducting polymer. In addition, the molar extinction coefficient (absorptivity) of the film visibly changed after modification by Fe^2+^/TPy. This increase is likely due to both the increase in polymer conjugation as well as incorporation of Fe^2+^/TPy into the polymer resulting in complex formation.

**Fig. 4:**
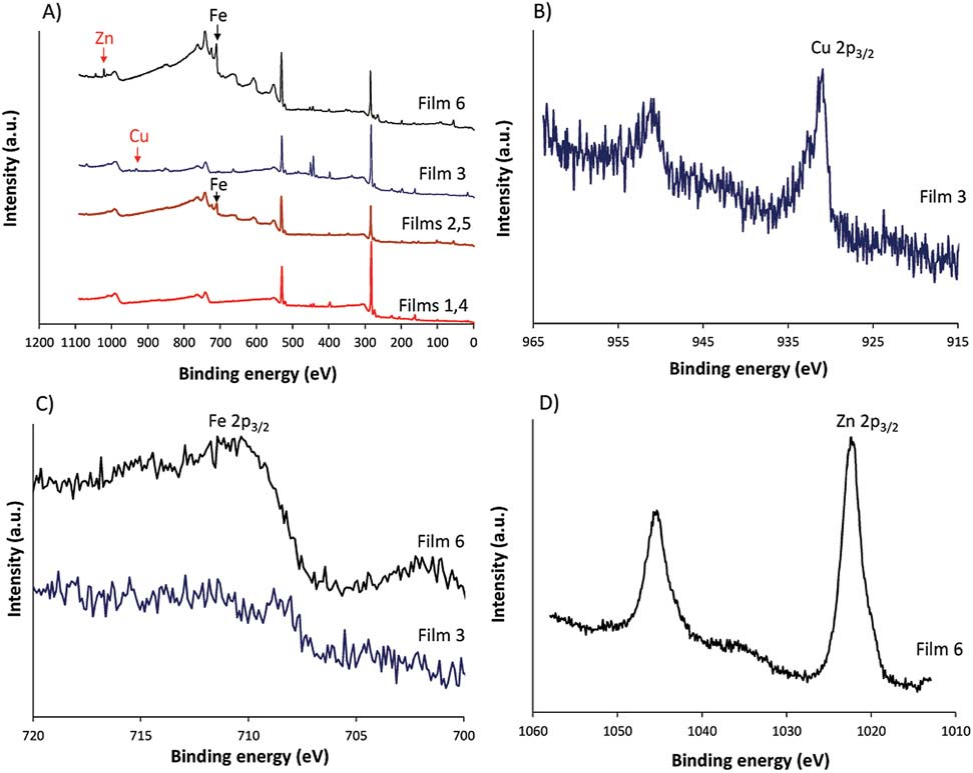
(A) XPS survey spectra of all films, (B) Cu 2p core level spectra of film 3, (C) Fe 2p core level spectra of films 3 and 6 and (D) Zn 2p core level spectra of film 6.

XPS and contact angle measurement of the surface of films before and after modifications were performed and the results are given in Fig. 4 and Table 1. The XPS analysis confirmed the presence of iron in the modified film (Fig. 4A). The contact angles before and after modification by Fe^2+^ and Fe^2+^-TPy did not differ.

**Table 1:** Elemental composition and contact angle data for poly(TTPy), poly(TTPy)Fe, poly(TTPy)Cu and poly(TTPy)Zn films

No.	C%	N%	O%	S%	Fe%	Cu%	Zn%	*θ*_c_ degrees
1, 4	77.44	3.5	14.9	4.1	N	—	—	104 ± 3.2
2, 5	68.9	1.6	26.0	1.9	1.6	—	—	99.7 ± 2.6
3	75.8	3.8	16.6	3.6	—	0.2	—	88.8 ± 1.8
6	63.7	1.8	29.0	1.7	3.5	—	0.3	85.5 ± 10.5

### Stability of the Fe[(TTPy)_2_]^2+^ complex in the presence of competing ions

**Fig. 5:**
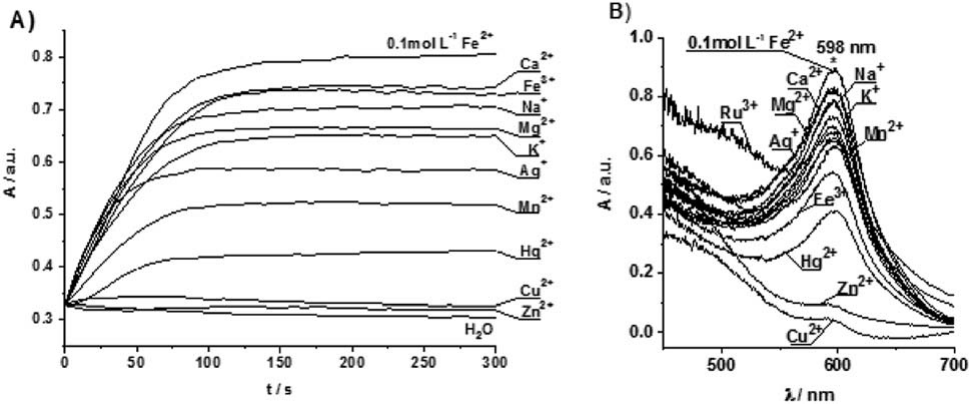
(A) Competing ion measurements performed by recording the absorbance at a single wavelength (598 nm) for Fe[(TTPy)_2_]^2+^ complex formation utilizing TPPy-SM deposited on the paper substrates in the solution containing 0.1 mol dm^−3^ of Fe^2+^ or 0.1 mol dm^−3^ of Fe^2+^ and 0.1 mol dm^−3^ of interfering ions (1 : 1): during 300 s measurement time. (B) Post measurement absorbance, recorded between 400 and 700 nm, performed in air on the dry paper strips.

Owing to the strong affinity of the terpyridine moiety towards various metal ions, the poly(TTPy)Fe film could be further modified. To this end, we undertook an additional study to select the most suitable metals ions to replace Fe^2+^ in the polymer film. In order to avoid the overlap of the broad absorption of the poly(TTPy) at 550 nm (Fig. 3B) with that of the absorption due to the polymer TTPy/Fe complex at 598 nm (Fig. 5B), we used monomer (TTPy) to investigate the formation of the Fe[(TTPy)_2_]^2+^ complex in the presence of seventeen different cations (Na^+^, K^+^, Mg^2+^, Ag^+^, Ca^2+^, Al^3+^, Sr^2+^, Zn^2+^, Fe^3+^, NH_4_^+^, Ba^2+^, Mn^2+^, Hg^2+^, Ni^2+^, Co^2+^, Cu^2+^ and Ru^3+^) with the kinetic procedures (protocols) developed in our previous study^4^ (Fig. 5). The TTPy monomer was incorporated into a polymer membrane on paper as to mimic the solid environment of the polymer. As shown by both measurement protocols (see Experimental section and ref. 4), the Cu^2+^ and Zn^2+^ ions yielded the lowest absorbances of TTPy/Fe complex formation, indicating that these two metal ions were competing for complex formation with Fe^2+^, this suggested that Cu^2+^ and Zn^2+^ ions could be a good candidates for decomplexing Fe from the poly(TTPy)Fe films.

### Modification of poly(TTPy) with Cu^2+^ and Zn^2+^

The poly(TTPy)Fe was further modified by Cu^2+^ and Zn^2+^ to obtain poly(TTPy)Cu and poly(TTPy)Zn films with different spectroelectrochemical properties. The electrochemical and optical properties of poly(TTPy)Cu (film 3) shown in Fig. 3A and B and poly(TTPy)Zn (film 6) in Fig. 3C and D. The oxidation potential of poly(TTPy)Cu was similar to that of unmodified poly(TTPy) (0.75 V) while the oxidation potential of poly(TTPy)Zn was 0.6 V. In both cases, loss in conjugation of the polymeric film would account for the shift in the oxidation potential towards higher potentials. This decrease in polymer conjugation was also observed in UV-VIS spectra, where the absorption spectra from poly(TTPy) decreased. The absorbance peak maximum for the poly(TTPy)Cu was 568 nm, 7 nm less than that of poly(TTPy)Fe. For the film modified by Zn^2+^ and Zn^2+^-TPy, the absorbance peak maximum for the new material was recorded at 583 nm, 8 nm more than that of poly(TTPy)Fe. Both modifications resulted in more hydrophilic films compared to pure poly(TTPy) and poly(TTPy)Fe as evidenced by the contact angle data in Table 1. Moreover, XPS analysis (Table 1) revealed that for the poly(TTPy)Cu film, all the Fe^2+^ was exchanged by Cu^2+^, while in the case of poly(TTPy)Zn, only a partial exchange, at least at the surface was observed. The electrochemical capacitance of the poly(TTPy)Zn film was similar to poly(TTPy)Fe. However, the electrochemical capacitance of poly(TTPy)Cu decreased dramatically compared to that of poly(TTPy)Zn or poly(TTPy)Fe, providing the information that introduced Cu^2+^ influenced the integrity of the modified film, thus reducing its electroactivity. This fact indicated the loss in the conjugation of the modified film. The poly(TTPy)Zn film remain the integrity of the poly(TTPy) as the peak maximum of the conducting polymer, around 550 nm remain. The major difference between poly(TTPy)Cu and poly(TTPy)Zn was the solvent used, where for Zn^2+^ and Cu^2+^ modifications 1 : 1 ACN : H_2_O and ACN were applied, respectively. In the presence of ACN partial release of Cu^2+^ may occur that further on acts as oxidizing agent for the conducting polymer film. This phenomenon was investigated and reported in Cu^2+^/ACN polythiophene aggregates chemistry.^35^ Moreover, the XPS core level spectra (Fig. 4B) revealed that the binding energy of Cu 2p_3/2_ in poly(TTPy)Cu was ∼932, which corresponds to Cu(i) and/or Cu(0).^36,37^ These data indicated that redox reactions may have influenced on the stability of the system and caused reduction of Cu^2+^ on the surface of film. The binding energy of Zn 2p_3/2_ in poly(TTPy)Zn was ∼1022 eV (Fig. 4D), which is attributed to Zn(ii) species.^38^ The binding energy of Fe 2p_3/2_ in this film was ∼711 eV and could be ascribed to both Fe(ii) and Fe(iii) states.^37^

**Fig. 6:**
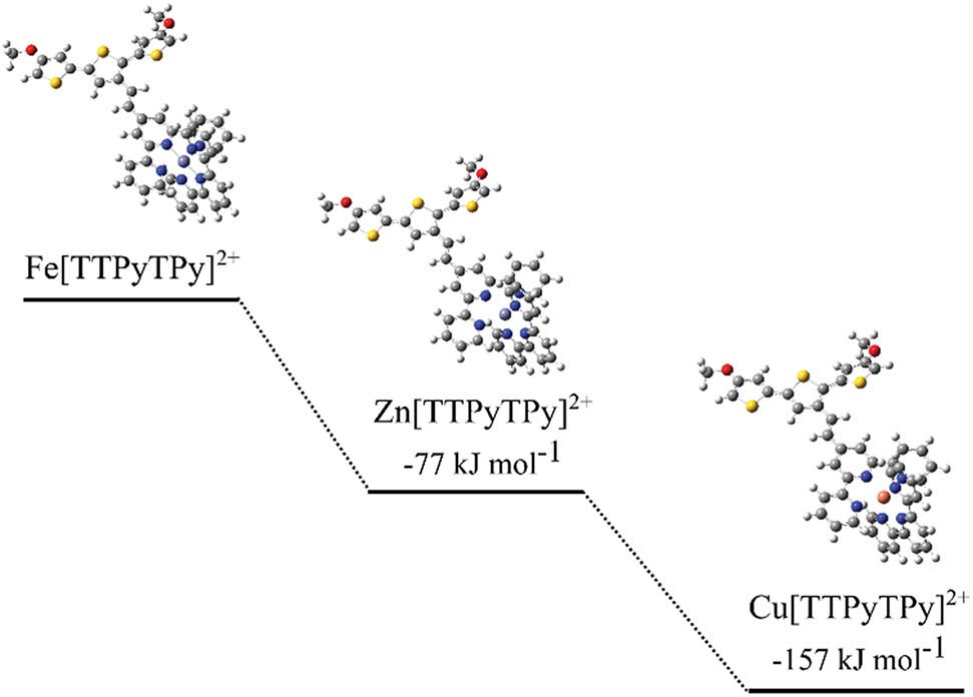
Structural representations of modelled TTPy complexes and calculated Δ*G* values, relative to Fe[TTPyTPy]^2+^.

DFT calculations were carried out to investigate metal exchange observed experimentally by cyclic voltammetry and optical spectroscopy. Formation energies (Δ*G*) were calculated for each metal complex using the chemical stoichiometry shown in eqn (1)–(3). The Δ*G* values were compared between cations to give relative binding preferences (Fig. 6).

The calculations show a relative preference for Zn^2+^ (−77 kJ mol^−1^) and Cu^2+^ (−157 kJ mol^−1^) complexes compared to the Fe^2+^ equivalent. This supports the experimentally observed exchange of Fe^2+^ to Cu^2+^ and Zn^2+^. The Cu^2+^ and Fe^2+^ results are consistent with binding constants for Cu^2+^ and Fe^2+^ with the nitrogen binding ligand 2,2-bipyridine.^39–41^ A difference in energy between the cations is likely due to N–metal binding affinities and preferential binding geometries. The predicted N–metal distances were explored in more detail in Table 2. Some differences in the geometries between the metal complexes were observed. For example, a ‘clamped’ geometry was predicted for Fe[TTPyTPy]^2+^ with short N–Fe bonds (2.00 Å) and a distorted terpyridine structure (5, 4′, 5′′ bond angle of 82.9° *versus* 93.4° for bare terpyridine). Zn[TTPyTPy]^2+^ exhibits the longest N–M bonds at 2.22 Å, while 2.17 Å is predicted for Cu[TTPyTPy]^2+^ with the least ligand angle deformation. These results suggest that Cu^2+^ binding results in the least distortion of the TPy residue and may be more highly conjugated within the terpy residue. In contrast, Fe^2+^ binding results in the largest distortion, which goes some way to support the experimental binding trends (Cu^2+^ > Zn^2+^ > Fe^2+^).

**Table 2:** Average predicted bond lengths and ligand angles. Ligand angles were averaged between the 5, 4′ and 5′′ carbons on both TTPy and TPy

	N–M bond length/Å	5, 4′, 5′′ bond angle/°
Fe[TTPyTPy]^2+^	2.00	82.9
Zn[TTPyTPy]^2+^	2.22	88.0
Cu[TTPyTPy]^2+^	2.17	88.5

In accordance with the results for Cu^2+^ shown above, a reduced Cu^+^ cation was also considered. This is especially valid as Cu^+^ is more stable than Cu^2+^ in acetonitrile and reduction of Cu^2+^ may occur.^42^ Cu[TTPyTPy]^+^ complex were predicted to be less favorable compared to Cu[TTPyTPy]^2+^ (a difference of +35 kJ mol^−1^). The energy difference is thought to be due to the well-established preferential geometries of Cu^+^ and Cu^2+^. Cu^+^ favors a tetrahedral arrangement and Cu^2+^ favors a trigonal bipyramidal arrangement.^43,44^ The binding pockets of TPy_2_ and TPPyTPy are more trigonal bipyramidal like, hence are best suited to a Cu^2+^ cation. When considering Zn^2+^ and Fe^2+^ cations, Cu^+^ would be preferred (−45 and −122 kJ mol^−1^ respectively).

## Conclusions

The electropolymerization of a thin conducting polymeric film based on the repeating unit of (*E*)-4′-(2-(4,4′′-bisdecyloxy-2,2′:5′,2′′-terthiophen-3′-yl)ethenyl)-2,2′:6′,2′′-terpyridine (TTPy) was achieved by the application of cyclic voltammetry. Further on, the same polymeric material was found useful in tuning its electrochemical and optical properties by application of molecular architecture *via* surface and bulk modifications of the conducting film. The modification was done by different metal ions and their terpyridine complexes in various solvents (ACN and 1 : 1 ACN : H_2_O). The electrochemical and optical properties of the conducting polymer films changed dramatically, namely films modified by Fe^2+^ and Fe^2+^-TPy resulted, in comparison to pure poly(TTPy), in an increased electrochemical capacitance, better conjugation of the conducting polymer system and higher absorptivity of the obtained film. Further exchange of the Fe^2+^ to Zn^2+^ and Cu^2+^ resulted in different electrochemical and optical properties of the obtained modifications. The electrochemical capacitance of the Zn^2+^ modified conducting polymer was maintained while optical properties of the film changed, while the electrical capacitance of the Cu^2+^modified film significantly decreased indicating deterioration of the conducting polymer structure.

The surface and bulk modification of the polymeric film as demonstrated here may lead to the application of this methodology to obtain a material with tunable optical and electrochemical properties. Such modification may be obtained, in poly(TTPy), by application of different metal ions and various ligands equipped with terpyridine functionality as well as application of various solvents during the modification. Such materials may have application in changeable and switchable surfaces and intelligent sensors.

## Conflicts of interest

There are no conflicts to declare.

## Supplementary Material

## References

[cit1] Manca P., Pilo M. I., Sanna G., Zucca A., Bergamini G., Ceroni P. (2011). Chem. Commun..

[cit2] Storrier G. D., Colbran S. B., Hibbert D. B. (1995). Inorg. Chim. Acta.

[cit3] Wagner M., Lisak G., Ivaska A., Bobacka J. (2013). Sens. Actuators, B.

[cit4] Lisak G., Wagner K., Wagner P., Barnsley J. E., Gordon K. C., Bobacka J., Wallace G. G., Ivaska A., Officer D. L. (2016). Synth. Met..

[cit5] Mondal P. C., Kantor-Uriel N., Mathew S. P., Tassinari F., Fontanesi C., Naaman R. (2015). Adv. Mater..

[cit6] Collis G. E., Burrell A. K., Scott S. M., Officer D. L. (2003). J. Org. Chem..

[cit7] Field J. S., Haines J., Lakoba E. I., Hal Sosabowski M. (2001). J. Chem. Soc., Perkin Trans. 1.

[cit8] Collis G. E., Burrell A. K., Officer D. L. (2001). Tetrahedron Lett..

[cit9] Schubert U. S., Eschbaumer C. (2002). Angew. Chem., Int. Ed..

[cit10] R Meier M. A., Lohmeijer B. G. G., Schubert U. S. (2003). J. Mass Spectrom..

[cit11] Jeong B.-S., Choi H.-Y., Kwak Y.-S., Lee E.-S. (2011). Bull. Korean Chem. Soc..

[cit12] Machan C. W., Adelhardt M., Sarjeant A. A., Stern C. L., Sutter J., Meyer K., Mirkin C. A. (2012). J. Am. Chem. Soc..

[cit13] Eloi J.-C., Chabanne L., Whittell G. R., Manners I. (2008). Mater. Today.

[cit14] Shunmugam R., Gabriel G. J., Aamer K. A., Tew G. N. (2010). Macromol. Rapid Commun..

[cit15] Qiu D. F., Zhao Q., Bao X., Liu K., Wang H., Guo Y., Zhang L., Zeng J., Wang H. (2011). Inorg. Chem. Commun..

[cit16] Jochum F. D., Brassinne J., Fustin C.-A., Gohy J.-F. (2013). Soft Matter.

[cit17] Dong R., Zhou Y., Huang X., Zhu X., Lu Y., Shen J. (2015). Adv. Mater..

[cit18] Sakamoto R., Wu K.-H., Matsuoka R., Maeda H., Nishihara H. (2015). Chem. Soc. Rev..

[cit19] Fan C., Ye C., Wang X., Chen Z., Zhou Y., Liang Z., Tao X. (2015). Macromolecules.

[cit20] Grant D. K., Officer D. L. (2005). Synth. Met..

[cit21] Kimura M., Horai T., Hanabusa K., Shirai H. (1998). Adv. Mater..

[cit22] Ciesielski A., Palma C.-A., Bonini M., Samori P. (2010). Adv. Mater..

[cit23] Collis G. E., Campbell W. M., Officer D. L., Burrell A. K. (2005). Org. Biomol. Chem..

[cit24] Gambhir S., Wagner K., Officer D. L. (2005). Synth. Met..

[cit25] Grant D. K., Jolley K. W., Officer D. L., Gordon K. C., Clarke T. M. (2005). Org. Biomol. Chem..

[cit26] Wagner K., Crowe L. L., Wagner P., Gambhir S., Partridge A. C., Earles J. C., Clarke T. M., Gordon K. C., Officer D. L. (2010). Macromolecules.

[cit27] Wagner K., Byrne R., Zanoni M., Gambhir S., Dennany L., Breukers R., Higgins M., Wagner P., Diamond D., Wallace G. G., Officer D. L. (2011). J. Am. Chem. Soc..

[cit28] Horvath R., Gordon K. C. (2010). Coord. Chem. Rev..

[cit29] Frisch M. J. , et al., in Gaussian, Inc., Wallingford, 2009, p. 09

[cit30] Sezai Sarac A., Evans U., Serantoni M., Clohessy J., Cunnane V. J. (2004). Surf. Coat. Technol..

[cit31] Kim D., Yoon J., Won M., Shim Y. (2012). Electrochim. Acta.

[cit32] Siebert R., Akimov D., Schmitt M., Winter A., Schubert U. S., Dietzek B., Popp J. (2009). ChemPhysChem.

[cit33] Jadamiec M., Lapkowski M., Officer D. L., Wagner P., Gordon K. C. (2009). Int. J. Nanotechnol..

[cit34] Milani A., Brambilla L., Del Zoppo M., Zerbi G. (2007). J. Phys. Chem. B.

[cit35] Gato H., Yashima E. (2002). J. Am. Chem. Soc..

[cit36] Donnelly V. M., Gross M. E. (1993). J. Vac. Sci. Technol. A.

[cit37] Folmer J. C. W., Jellinek F. (1980). J. Less-Common Met..

[cit38] Moulder J. F. , Stickle W. F., Sobol P. E. and Bomben K. D., Handbook of X-ray Photoelectron Spectroscopy: A reference book of standard spectra for identification and interpretation of XPS data, Physical Electronics Division, Perkin-Elmer Corporation, 1992

[cit39] McBryde W. A. E. , Critical Evaluation of Equilibrium Constants in Solution: Stability Constants of Metal Complexes, Elsevier, 2013

[cit40] Hazra D. K., Lahiri S. C. (1975). Anal. Chim. Acta.

[cit41] Pantani F. (1967). Ric. Sci..

[cit42] Kratochvil B., Zatko D. A., Markuszewski R. (1966). Anal. Chem..

[cit43] Fraser M. G., Salm H., Cameron S. A., Barnsley J. E., Gordon K. C. (2013). Polyhedron.

[cit44] Barnsley J. E., Scottwell S. Ø., Elliott A. B., Gordon K. C., Crowley J. D. (2016). Inorg. Chem..

